# Prognosis of 18 H7N9 Avian Influenza Patients in Shanghai

**DOI:** 10.1371/journal.pone.0088728

**Published:** 2014-04-02

**Authors:** Shuihua Lu, Tao Li, Xiuhong Xi, Qingguo Chen, Xuhui Liu, Binxing Zhang, Jiaxian Ou, Jie Liu, Qin Wang, Biao Zhu, Xinian Liu, Chunxue Bai, Jieming Qu, Hongzhou Lu, Zhiyong Zhang, Yuanlin Song

**Affiliations:** 1 Department of Tuberculosis, Shanghai Public Health Clinical Center, Shanghai, China; 2 Department of Pulmonary Medicine, Zhongshan Hospital, Fudan University, Shanghai, China; 3 Department of Surgery, Zhongshan Hospital, Fudan University, Shanghai, China; 4 Department of Pulmonary Medicine, Huadong Hospital, Fudan University, Shanghai, China; 5 Department of Pulmonary Medicine, Zhongshan Hospital, Qingpu Branch, Shanghai, China; PLOS, United Kingdom

## Abstract

**Purpose:**

To provide prognosis of an 18 patient cohort who were confirmed to have H7N9 lung infection in Shanghai.

**Methods:**

Patients' history, clinical manifestation, laboratory test, treatment strategy and mortality were followed and recorded for data analysis.

**Results:**

A total of 18 patients had been admitted into Shanghai Public Health Clinical Center from April 8^th^ to July 29, 2013. 22.2% of the patients were found to have live poultry contact history and 80% were aged male patients with multiple co-morbidities including diabetes, hypertension and/or chronic obstructive pulmonary disease (COPD). This group of patients was admitted to the clinical center around 10 days after disease onset. According to laboratory examinations, increased C reactive protein (CRP), Procalcitonin (PCT), Plasma thromboplastin antecedent (PTA) and virus positive time (days) were indicative of patients' mortality. After multivariate analysis, only CRP level showed significant prediction of mortality (P = 0.013) while results of prothrombin time (PT) analysis almost reached statistical significance (P = 0.056).

**Conclusions:**

H7N9 infection induced pneumonia of different severity ranging from mild to severe pneumonia or acute lung injury/acute respiratory distress syndrome to multiple organ failure. Certain laboratory parameters such as plasma CRP, PCT, PTA and virus positive days predicted mortality of H7N9 infection and plasma CRP is an independent predictor of mortality in these patients.

## Introduction

The first H7N9 patient was identified in Shanghai on 31 March 2013 to commence the disease outbreak which resulted in a total of 132 confirmed cases across China with 43 deaths [1], plus two more recently identified new cases in Beijing and Guangzhou. Aside from fulminant pneumonia, respiratory failure and ARDS [2], H7N9 was characterized by extrapulmonary manifestation such as rhabdomyolysis and encephalopathy [2]–[4]. In an epidemiological study involving 82 patients, H7N9 was found to prevail in elderly male with substantial co-morbidities and historical livestock exposure or poultry industry engagement [5]. In Shanghai where the infection was first recognized, an emergency protocol was established to combat the infection. Till July 29, 2013, a total of 33 cases have been identified, among which 17 died and 16 were clinically cured, the gross mortality being 51.5%. Of 18 patients who were recruited into the Shanghai Public Health Clinical Center for intensive treatment, 6 of them died and all others were discharged from the hospital, the mortality being 33.3%.

The H7N9 infection reminded the public of precedent H5N1 and H1N1 infections which had impacted the world with high infection and mortality. Classified as type A influenza viruses under the RNA viral family Orthomyxoviridae, Their nomenclature is determined by their subtypes of hemagglutinin (HA) and neuraminidase (NA) molecules. Rapid evolution and variations in their antigenicity, pathogenicity and host specificity determine their capability of avian and mammalian infections. More importantly, the yield of a novel HA molecule via genetic reassortment or inter-species transmission may result in an influenza pandemic. Gao et al. has performed sequencing analyses in the H7N9 viruses isolated from respiratory specimens of 3 patients [2]. According to the study, all genes of the viruses were of avian origin and 6 internal genes had originated from H9N2 viruses. Specifically, substitution Q226L (H3 numbering) at the 210-loop in the HA gene of 2 viruses; a T160A mutation at the 150-loop in the HA gene of all viruses and deletion of 5 amino acids in the NA stalk region of all viruses have been identified.

Since the infection began, the pathogenesis, virology, clinical course and treatment of H7N9 have been elaborated to improve our understanding of the virus. In spite of the endeavor, none of the studies have elucidated the prognosis of H7N9 infection. Since April, 18 confirmed cases have been admitted to the intensive care unit of Shanghai Public Health Clinical Center for treatment. We collected detailed medical records and analyzed the patients for clinical presentation and laboratory tests. We also compared the information with previous H5N1 and H1N1 infections in order to provide indications for influenza diagnosis, differentiation and prognosis.

## Materials and Methods

### Patients and Study Design

The study was approved by the institutional review board of Shanghai Public Health Clinical Center with written informed consent obtained from all patients or their surrogates. 18 patients with positive H7N9 viral isolation from throat swab were transferred to the intensive care unit of Shanghai Public Health Clinical Center and recruited to the study. Detailed medical history was collected while laboratory tests and imaging examinations were performed. Standard care, antiviral therapy, antibiotic therapy and assisted ventilation (when required) were administered to the patients as specified. Each patient was followed for a maximum of 15 weeks in a prospective cohort from admission till death/discharge to compare their medical history, clinical manifestation, laboratory tests, imaging examinations and clinical outcomes. Patients were grouped into either the survived or non-survived group depending on their clinical outcome at the end of follow-up.

### Treatment Strategy

All recruited patients received antiviral and antibiotic therapies to alleviate infection and standard care to stabilize vital signs. An antiviral therapy of Tamiflu 75 mg bid was administered to all patients from the first day of admission till (i) negative twice for throat swab test. An antibiotic therapy of levofloxacin 0.4–0.6 g qd was given for up to 7–10 days but altered to piperacilin/sulbactum and levofloacxin under (i) elevations in total white blood cell count and percentage of neutrophils; (ii) high body temperature; and (iii) purulent sputum. Antibiotic therapy was terminated under (i) body temperature <37.5°C; (ii) absence of purulent airway secretion; and (iii) stable or partial resolution of lung infiltration. Susceptible antibiotics were administered accordingly if bacterial culture had been positive in tracheal inspiration or sputum. Methylpredisolone 40 mg qd-bid was intravenously administered under (i) increased lung filtration in <12 hours; (ii) progressive deterioration of hypoxia; and (iii) hemodynamic instability. The regimen stopped after 3–5 days but extended under unimproved lung infiltration and high plasma level of C-reactive protein (CRP). Albumin 10 g, followed by frosemid 20 mg, was intravenously administered daily if serum albumin level <28 g/L. Gamma globulin 10 g qd was administered till recovery. The amount of fluid supplemented (inlet) was 500 ml more than that of the outlet. Nutrition and energy were provided with placement of a nasogastric tube or delivery of total parenteral nutrition via inline central venous catheter. Inhalation therapies (corticosteroid, Ventolin) were provided based on physician's determination of patient's respiratory symptom, physical sign and laboratory results. Oxygen inhalation (1–3 L/min) was adopted to maintain the minimum SaO_2_ at 90%. It was adjusted accordingly if blood gas analysis showed that PaO_2_<60 mmHg. Non-invasive ventilation was added if respiratory rate >25/min. Invasive ventilation was applied via BIPAP mode (PEEP 8–20 cmH2O, PIP 15–20 cmH_2_O, RR 8–15) or SIMV +PSV mode (RR 2–8/min, PEEP 8–20 cmH2O, PSV 15–20 cmH_2_O) when patients presented respiratory distress and unstable hemodynamic. Fraction of inspiration of oxygen (F_i_O_2_) varied from 30% to 100% to maintain P_a_O_2_ at minimum of 60 mmHg. In addition to the existing ventilator support, Extra-Corporeal Membrane Oxygenation (ECMO) (VV mode; instrument from Marquette) were performed by an experienced anesthesiologist via femoral and internal jugular vein cannulation if oxygenation (PaO_2_/FiO_2_)<80 and could not be corrected within 24 hours. Specifically, blood flow was set at 3.5–4 L/min to ensure the minimum SaO_2_ at 91%. Activated clotting time (ACT) was routinely checked every 2 hours within the 24 hours of ECMO application. Either heparin or platelet was added to keep ACT between 180–220 s. If platelet count <30×10^9^/L, 1–2 platelet units would be supplied till platelet count increased to no less than 30×10^9^/L.

### Definition of Severe Pneumonia and ARDS

The patients were diagnosed to have severe pneumonia and/or ARDS was based on 2007 ATS/IDSA guidelines [6] and 2011 Berlin Definition [7].

### Examinations

All recruited patients were subjected to inpatient laboratory examinations upon admission. Among such, standard blood chemistry (e.g. liver function, kidney function, electrolytes, myocardial enzymes and amylase) was repeated daily; routine blood test, inflammatory indicators (Procalcitonin *PCT* and CRP), coagulation function (D-dimer, Thrombin time *TT*, Activated partial thromboplastin time *APTT*, Prothrombin time *PT*, and Plasminogen activator inhibitor-1 *PAI-1*) and pathogenic detection (in blood, tracheal aspiration, urine or other body fluids) were re-examined when suspected of local infection, blood dissemination or sepsis. Bedside chest X-ray was taken daily and every 2 days respectively under ECMO and invasive ventilation. Chest-CT was performed every 2–3 days among mild to moderate phenotypes.

### Statistical Analysis

We used descriptive statistics to summarize the characteristics of recruited patients and SPSS version 17.0 (SPSS, Inc., Chicago IL) to analyze the data. Mean values were respectively computed for the routine blood test, blood chemistry and inflammatory indicators of all recruited patients. If data had normal distribution, Student's t test was applied to compare the results between survived and non-survived groups; otherwise, the Wilcoxon Rank-Sum test was applied to conduct statistical analysis between two groups. P-values<0.05 (two-sided probability) were considered statistically significant. Logistic regression was applied to test the prediction value of certain laboratory indications by univariate and multivariate analysis. Fisher's exact probability test was conducted to examine the association between patients' underlying diseases and prognosis.

## Results

### Patient Characteristics

Among 18 patients, 6 died and the others were discharged for follow up. Table 1 illustrates the characteristics of 18 recruited H7N9 patients. Their mean age was 69.2±8.6 years. 14 subjects were male while 4 others were female. 4 patients (22.2%) admitted, 13 patients (72.2%) denied and 1 patient (5.6%) was unsure about close contact with live poultry prior infection. All patients presented substantial co-morbidities including hypertension (8; 44.4%), diabetes mellitus (4; 22.2%), cardiovascular disease (5; 27.8%), liver diseases (2; 11.1%) and chronic obstructive pulmonary disease (COPD) (2; 11.1%). For the initial symptoms of recruited patients upon disease onset. 10 (55.6%) patients presented fever and cough; 4 (22.2%) presented only fever; 2 (11.1%) started merely with cough; 1 (5.6%) felt fatigued and 1 (5.6%) presented a combination of fever and fatigue (1; 5.6%). The mean time between initial symptoms and hospital admission or diagnosis was 9.8±1.6 and 9.3±1.7 days respectively. The initial symptom was similar to those of H1N1, H5N1 except that H7N9 patients did not complain of conjunctivitis (Table 2).

**Table 1:** Patients' characteristics.

Items	N = 18	%
Age	69.2±8.6	/
Gender(M/F)	14/4	77.8/22.2
Close contact history of birds(Y/N/Unknown)	4/13/1	22.2/72.2/5.6
Mild case	8	44.4
With non-invasive ventilation	4	22.2
Invasive ventilation	3	16.7
Invasive ventilation+ECMO	3	16.7
ARDS (died)	5(4)	26.7(22.2)
Severe pneumonia	6(5)	33.3(26.7)
**Underlying Diseases** *		
Hypertension	8	44.4
Diabetes Mellitus	4	22.2
Cardiovascular Disease	5	27.8
Liver Diseases	2	11.1
COPD	2	11.1
**Onset**		
Initial Symptoms		
Fever	18	100
Cough	15	83.3
Fatigue	3	16.7
Progressive breathless	6	33.3
Hemoptysis	1	5.6
Time between onset of symptoms and admission(days)	9.8±1.6	/
Time between onset of symptoms and diagnosis(days)	9.3±1.7	/
**Treatment**		
On Tamiflu	18	100.0
Time between onset of symptoms and treatment(days)	7.9±1.6	/
Duration(days)	11.3±1.2	/
Corticosteroids	14	77.8
Time between onset of symptoms and treatment(days)	10.4±1.9	/
Duration(days)		/
Antibiotics	18	100.0
Time between onset of symptoms and treatment(days)	5.3±1.7	/
Duration(days)		/
**Mortality at 28 days**	5	27.8
**Mortality at week 6**	6	33.3

*: 1 patient had cochlea disease; 1 patient had history of breast cancer(after surgery); 1 patient had rheumatoid arthritis; 1 had hypothyroidism; 1 patient had cholecystolithiasis;1 patient had gout; 1 patient had facial paralysis and 2 of them had benign prostate hyperplasia. Data was Mean ± SE. Y/N, Yes/No; ECMO, Extra-Corporeal Membrane Oxygenation; ARDS, Acute respiratory distress syndrome; COPD, Chronic obstructive pulmonary disease.

**Table 2:** The Comparison between H5N1, H1N1 and H7N9.

	H5N1	H1N1	H7N9
**Year of outbreak**	1997, 2003	2009	2013
**Major sites**	Hong Kong, Europe, Africa, Southeast Asia	Mexico, U.S.	China, Taiwan
**Epizootic**	• Widespread fatal poultry outbreaks	Nil	• Void of large-scale fatal poultry outbreaks
	• Spread panzootically in poultry and wild-bird populations [10]		• Confined to live-bird markets and circumscribed poultry foci [10]
**Occurrence**	447 cases & 263 deaths globally as of 21 December 2009 [11]	11034 cases & 85 deaths in 41 countries as of 21 May 2009 [12]	132 cases & 43 deaths in China; 1 case in Taiwan as of 30 June 2013 [1]
**Susceptible population**	• People in close contact with live poultry [12]–[15] or contaminated environment [17]	Children & young adults without underlying diseases [18]	Elderly male with substantial co-morbidities & historical poultry exposure [5]
**Clinical manifestation**	• Viral pneumonia		
	• ARDS		
	• Extrapulmonary symptoms: renal failure, multiple organ failure, CNS involvement, etc.		
	• Symptoms usually develop 2–4 days after poultry exposure	• Upper respiratory tract infection	• Viral replication in upper & lower airways, virulent in the lower
	• Viral replication in lower airway [16], [19]	• Secondary bacterial pneumonia	
		• Asians seemed to have lower rate of severe disease [20]	
**Laboratory tests**	• Lymphopenia (inverted ratio of CD4+ T cells to CD8+ T cells)	• Changes in WBC count according to disease severity	• Early drop of WBC followed by restoration
	• Hypercytokinemia	▪ Mild: Great drop of WBC	• Thrombocytopenia
	• Hyperchemokinemia Thrombocytopenia	▪ Severe: non-significant drop of WBC	• Abnormal liver function: increased levels of AST, ALT, LDH & decreased albumin
	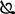 Increased serum levels of hepatic transaminases	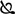 Increased neutrophil count at acute phase of severe cases	• Increased levels of myocardial enzymes (CK & CK-MB)
	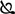 Increased plasma creatinine [13], [14], [16], [19], [21]–[24]	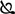 Decreased peripheral lymphocytes, CD3, CD4, CD8 and B cells in acute phase and recovered in recovery phase	• Increased levels of CRP & amylase
		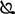 Decreased NK and NKT cell counts at acute phase of severe cases [25]	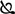 Abnormal coagulation or fibrinolysis: D-Dimer increases
		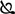 Persistent hypercytokinemia in severe cases [26]	
		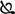 Lower serum IgG2 level might associate with disease severity, especially in pregnancy [27], [28]	
		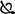 Increased serum levels of hepatic transaminases (AST, ALT, GGT & LDH) [29]	
		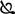 Elevated CRP [30]	
**Imaging**	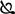 Extensive & often bilateral infiltration	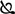 Bilateral/lobular infiltration: peripheral in adults & diffuse in children	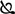 Patchy/lobular infiltration with rapid & progressive changes
	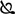 Lobular collapse	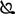 Generally bilateral consolidation & ground-glass opacity	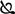 Bilateral ground-glass opacity and consolidation which may become white lungs
	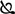 Focal consolidation	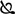 Possible signs of pulmonary emboli on CT [31]	
	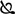 Air bronchograms		
**Treatment**	NA inhibitors (oseltamivir, zanamivir, peramivir) prevail over adamantanes as first-line therapy [25], [30].		

In compliance with the criteria stated in “Materials and Methods”, antiviral (Tamiflu) and antibiotic therapies were offered to all recruited patients while steroid therapy was provided to 14 (77.8%) patients. As stated in Table 1, considerable time lags existed between appearance of initial symptoms and administration of therapies. On average, antibiotics were given the earliest (5.3±1.7 d), followed by Tamiflu (7.9±1.6 d) and corticosteroids (10.4±1.9 d).

Of note, ARDS (P<0.01) and severe pneumonia (P<0.01) contributed to patient's mortality. Of 18 patients, 6 had severe pneumonia and 5 developed ARDS. 5 out 6 severe pneumonia patients died and 4 out of 5 ARDS patients died. It is therefore critical to manage ARDS and severe pneumonia in order to reduce mortality.

Besides, for the consideration of basic health conditions effect on the prognosis, we further analyzed the association between patients' clinical prognosis and their main underlying diseases including hypertension, diabetes mellitus, chronic cardiovascular diseases, chronic liver disease and chronic respiratory diseases (2 of these 18 patients had history of COPD) they suffered from before H7N9 infection, while no difference had been found between their outcomes and basic health conditions finally. (p-values were 0.44, 0.59, 0.44, 0.57, 0.57 in order). In this regard, health conditions prior to H7N9 infection might not make difference in clinical outcomes of these patients.

### Virus detection and multiple organ injuries from laboratory examination

#### Virus detection in different sites

**Figure 1:**
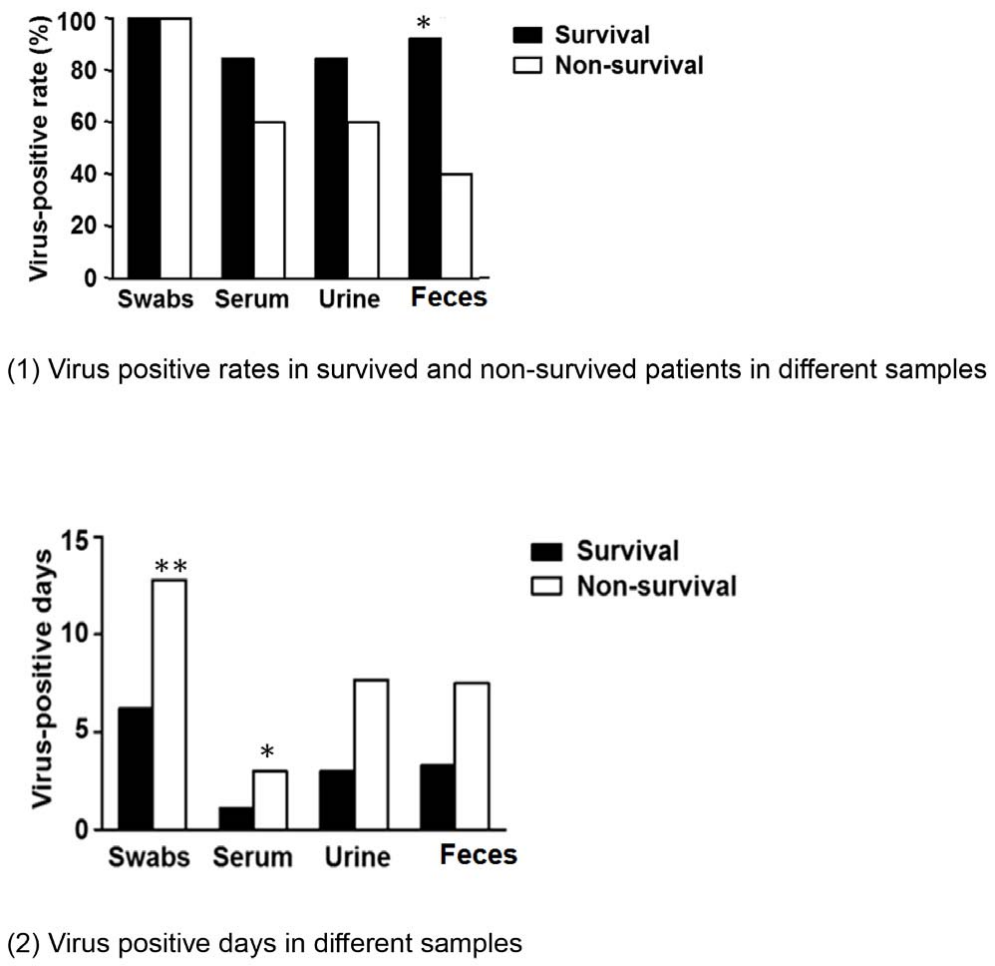
Virus detection in different samples. Figure A showed virus positive rates in survived and non-survived patients in different samples. All patients were virus positive in throat swab samples but displayed different virus positive rates in blood, urine, and feces samples. Figure B showed different virus positive time in survived and non-survived patients in different samples. In general, virus positive days were significantly prolonged in non-survived patients. (*, P<0.05; **, P<0.01).

**Figure 2:**
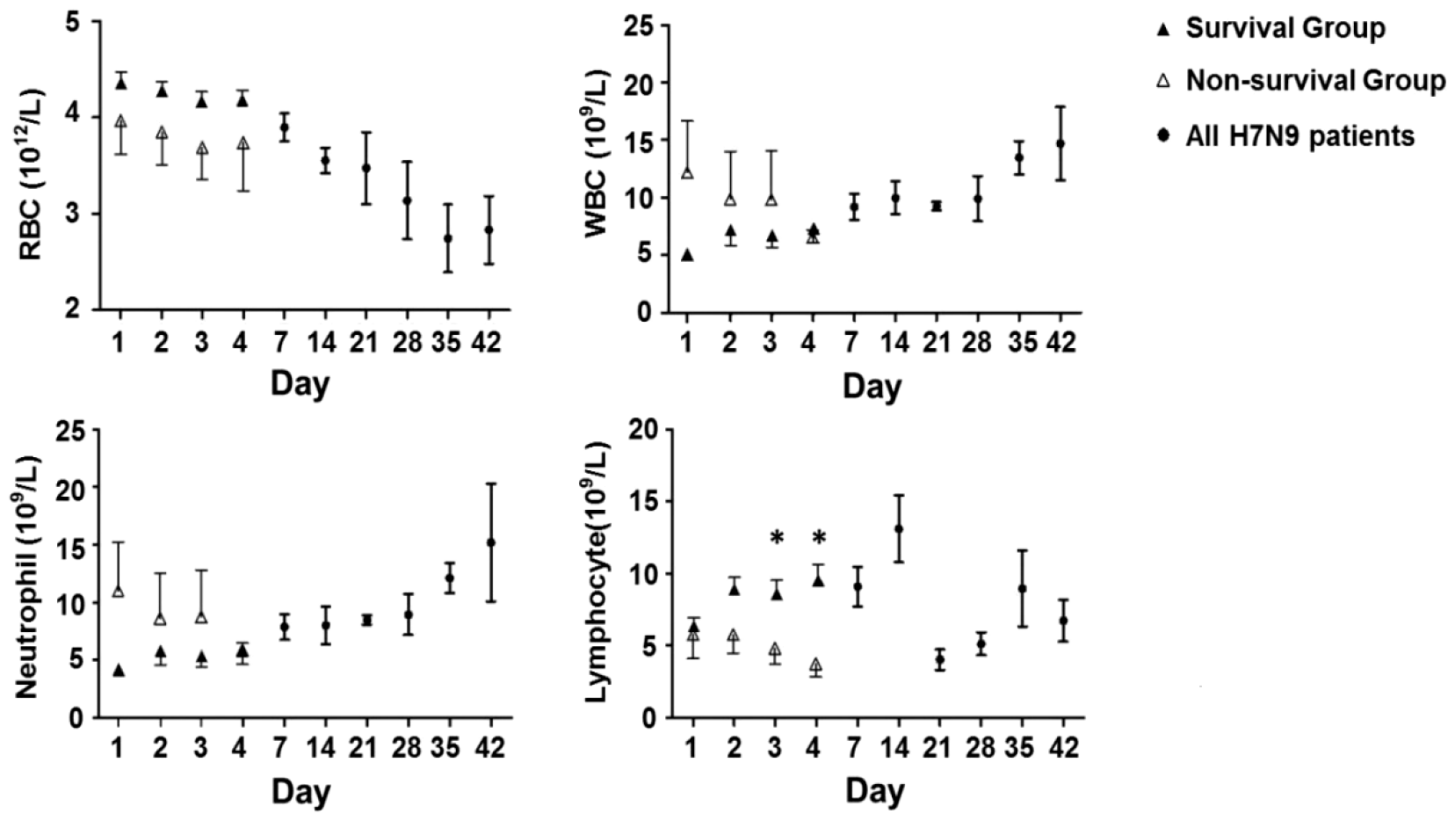
Routine blood test results. Panel A–D showed red blood cell, white blood cell, neutrophils and lymphocytes count changes following time. There was a clear trend of decreased red blood cell count (A), while white blood cell and neutrophils numbers were increased (B,C). There was a fluctuation of blood lymphocytes changes (D). There were more significant changes of blood cells in non-survived patients within 4 days after hospitalization, especially the lymphocyte count decreasing 3 and 4 days after admission in non-survived group. (*, P<0.05), data represents as median +/−25–75% CI. Open triangle, survival group; solid triangle, non-survived group; solid circle, all patients. RBC, red blood cell; WBC, white blood cell.

Positive virus detection in multiple sites confirmed viral spreading from upper airways to deep lungs and other organs (figure 1). The average virus positive days in different samples varied depending on sites. Correspondingly, the virus positive days of survived and non-survived patients lasted for 6.2 and 12.8 days in throat swab samples starting from the first day of confirmed diagnosis; 1.1 and 3.0 days in blood; 3.0 and 7.7 days in urine and 3.3 and 7.5 days in feces starting from admission (figure 1–2). Such results implied how virus positive time correlated with mortality and longer virus positive days could be indicative of worse outcome (figure 1–2). In this regard, significantly delayed virus clearance might be related to the mortality of non-survived patients.

#### Blood cell changes

Similar to previous report [5], H7N9 infection showed systemic organ injury involving systemic circulation, kidney, liver, pancreases and muscles. Erythrocytopenia, neutropenia, and lymphopenia are blood cell changes commonly seen in influenza patients at initial stage (table 2). Dynamic changes in mean red blood cell, white blood cell (WBC), neutrophils, and lymphocyte counts were observed among 18 recruited H7N9 patients within 7 weeks of hospital admission. A clear de-association of the above cell lines was also seen 4 days within admission, particularly for lymphocyte count in which significant difference was found between survived and non-survived patients on days 3 and 4 (P<0.05). In general, progressive decrease of mean red blood cell count, increased number of WBC and neutrophils and fluctuation of lymphocytes among the recruited patients suggested the possibilities of persistent anemia and infection over time as a result of complete cell line damage in virus infection and secondary bacterial infection.

#### Coagulation activities changes

**Figure 3:**
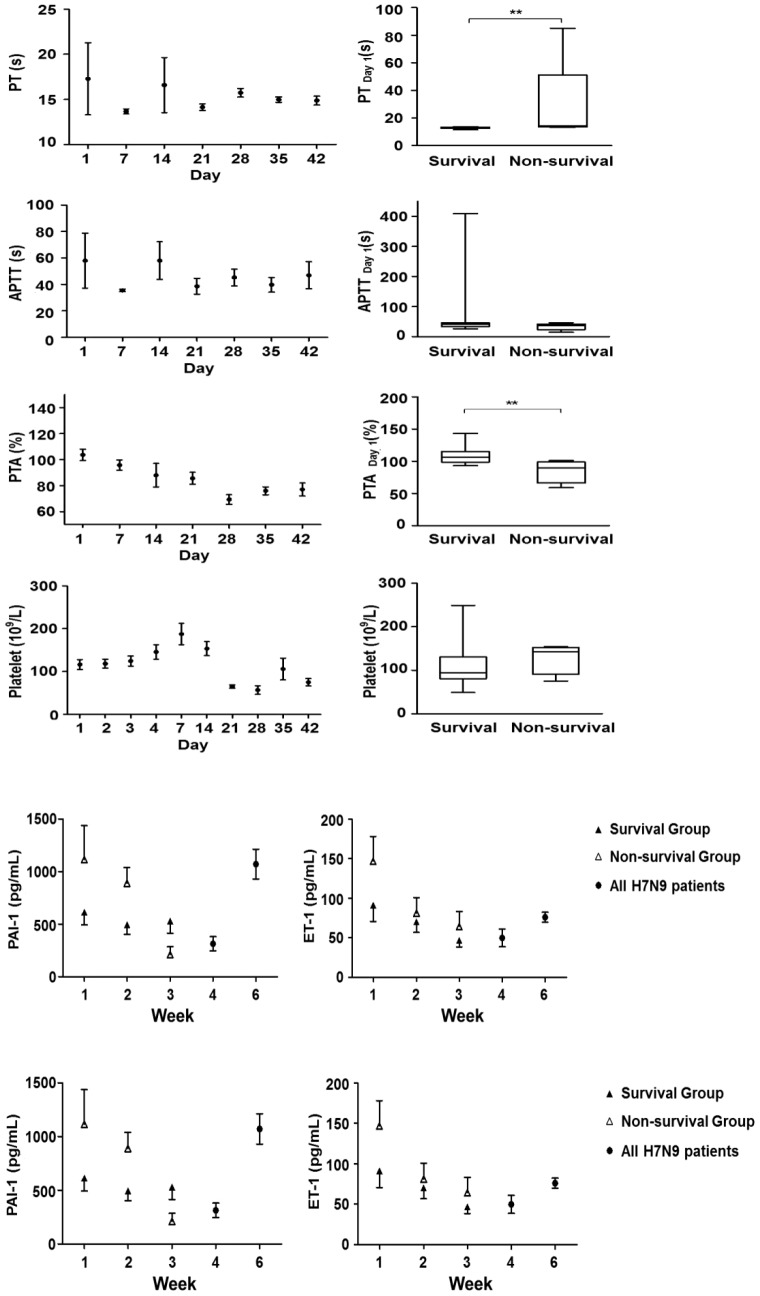
Blood coagulation activity and platelet counts results. PT, APTT, PTA and platelet were quantified daily 4 days within hospitalization then weekly after. For patients who had respiratory failure, these parameters were followed daily or every two days till discharge or death but data presented here was weekly data. There was a clear trend of platelet count increasing within 1 week after admission then dropped again. For hospitalization day 1, there was a significant increase of PT but decreased PTA in non-survived group compared to survived patients. There was no significant changes of APTT and platelet changes at hospitalization day 1. PT, Prothrombin Time; APTT, activated partial thromboplastin time; PTA, Prothrombin activity. Data represents as median +/−25–75% CI. (**, P<0.01).

High coagulation activity is a common finding in severe pneumonia [8]. To monitor dynamic changes in coagulation activity among survived and non-survived patients, PT, APTT, PTA, platelet and PAI-1 were measured at different time points (Figure 3).

Increased PT, significant decrease of PTA on the first day of admission (P<0.01) and progressive drop of PTA 3 weeks within admission, indicated the possibilities of increased extrinsic coagulation activities at early H7N9 infection as a result of massive endothelial cell damage. Elevations of serum PAI-1 and Endothelin-1 (ET-1) levels were also observed with the non-survived patients showing an trend of increase during the first week of admission, probably due to massive endothelial cell damage. PTA is generally considered indicative of possible liver injury, it was found to be significantly correlated with mortality just as increased PT did (Figure 3).

#### Blood chemistry changes

**Figure 4:**
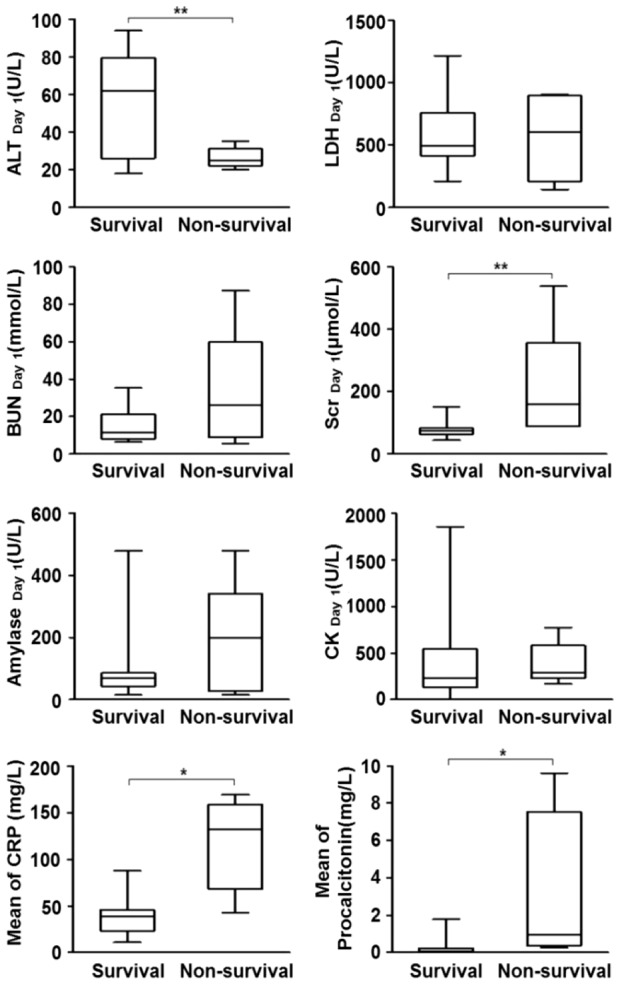
Blood chemistry in survival and non-survival patients. Serum ALT level was significantly lower in non-survived patients at hospital day 1. While for LDH, amylase, BUN and CK, the concentration in non-survived group was much higher than at in survived group. There was significant increase of serum creatinine, CRP and PCT in non-survived patients at hospital day 1. LDH, lactate dehydrogenase; ALT, Alanine aminotransferase; CRP, C-reactive protein; PCT, procalcitonin; BUN, Blood urine nitrogen; CK, creatine kinase; Scr: blood creatinine. Data represents as median +/−25–75% CI. (*, P<0.05; **, P<0.01).

To further evaluate changes in systemic organ function and alterations in liver, pancreas, kidney, muscles and inflammation status, selected organ enzymes and inflammation biomarkers were quantified at different time points. Figure 4 showed blood chemistry changes in survived and non-survived patients on day 1 of admission. Clearly, non-survived patients had higher Serum creatinine (Scr), CRP and PCT (P<0.05 for CRP and PCT, P<0.01 for Scr). Alanine transaminase (ALT) was higher in survived patients, the only parameters not consistent with other parameters (P<0.01). These changes suggested multiple organ injuries at the early stage of H7N9 infection and served as prognostic markers. The significant increase of CRP and PCT strongly suggested massive systemic inflammation and early bacterial infection.

A multivariate analysis was done to verify which parameters could be an independent predictors of mortality after balancing age, gender, initial severity of pneumonia, PT, PTA, ALT, Scr, CRP, PCT, and swab positive days. The results showed only CRP was independently associated with 3-month mortality while PT almost reached statistical significance (P = 0.056). This result suggested CRP, although a regular lab test in clinical practice, is still a useful predictor of long term mortality for patient who had H7N9 infection.

#### Bacterial culture

Bacterial infection might have been a serious complication in postal H7N9 infection since *A. Baumannii*. isolation was observed in three intubated patients. While serum PCT concentration on the first day of admission was predictive of mortality, early co-infection with bacteria was found to be associated with poor outcomes. In this regard, prevention of bacterial infection might improve patient outcomes and reduce days of hospitalization.

### ECMO application

Early application of ECMO may benefit patients with severe pneumonia and multiple organ failure. In our study, it was administered under severe conditions (P/F<80 for 24 hours) but all the 3 patients who had received the treatment died on an average survival of 50.7 days. Compared to the H1N1 episode, a higher mortality in this subgroup had been observed [9].

## Discussion

We have followed the clinical manifestation, laboratory examinations and prognosis of 18 H7N9 patients in Shanghai, China for 15 weeks starting from their first day of admission to Shanghai Public Health Clinical Center. In accordance with previous description [2], we concluded that elderly male with underlying co-morbidities and historical poultry exposure were susceptible to H7N9 infection. Most patients initially complained of fever and cough but did not receive timely treatment due to prolonged diagnosis and hospital admission. In-depth comparisons between precedent influenza pandemics will improve our capabilities in promptly managing future attacks.

We determined the virus positive days from the first day of confirmed diagnosis till when throat swab turned negative for twice.

The virus positive days correlated with patients' mortality: the longer the virus positive days, the higher the mortality; the sooner the virus was cleared, the higher the chance of recovery. This suggested how virus conversion and patients' immune response against H7N9 infection would probably determine the outcome of H7N9 infection.

The recruited H7N9 patients generally presented an early decrease of WBC count, thrombocytopenia, abnormal liver function (increased levels of AST, Lactate dehydrogenase *LDH* & decreased albumin) and increased levels of myocardial enzymes (CK- creatine kinase & its isoenzyme CK-MB), CRP, amylase and D-Dimer. While the changes in routine blood test were characteristic of viral infection, changes in blood chemistry suggested multiple organ damage which might manifest as extrapulmonary symptoms. Comparing the mean laboratory test results of survived and non-survived groups, the latter showed significantly lower red blood cell count and lymphocyte count but higher white blood cell count, neutrophil count, serum Blood urea nitrogen (BUN), Scr, CRP and PCT. No significant differences had been observed in mean serum amylase, ALT and LDH. The differences of PCT implied higher risks of mortality in patients with secondary bacterial infection. The 5 intubated patients with 3 of them had *A. Baumannii* isolation at different antibiotic-resistance suggested how post-viral infection with bacteria might contribute to patients' outcomes. In this regard, early intervention or effective prevention of *A. Baumannii* might improve patients' prognosis.

The treatment strategy of recruited patients consisted of antiviral therapy, antibiotic therapy, steroid regimen, assisted ventilation, fluid and nutritious supplement and other supportive therapies. Antiviral therapy, administered when throat swab indicated positive results, awaited an appropriate timing of delivery which would influence the efficiency of treatment. Of note, antiviral therapy continued till throat swab showed negative results twice and our experience demonstrated Tamiflu had been used in some patients for more than two weeks. It is therefore to keep in mind that throat swab results may not represent actual status of lower airways or blood. In this regard, pathogenic detection should be performed as soon as possible - the earlier a patient starts antiviral treatment, the better his chance of recovery.

With high resemblance, H7N9 infection reminded the public of precedent H5N1 and H1N1 pandemics which had resulted in significant morbidity and mortality. Belonging to the same family, these three kinds of influenza viruses bring about clinical conditions characteristic of high fever, cough, headache, malaise and inflammation of the upper and lower respiratory tract. In severe cases, complications like pneumonia, hemorrhagic bronchitis, multiple organ dysfunction, central nervous system involvement and death may result. Variations in epidemiology, clinical manifestation and auxiliary examinations between the three infections are listed in Table 2.

Of note, 3 patients received ECMO therapy but all of them died eventually from multiple organ failure. The averaged survival time was 50.7 days. Clearly these 3 patients received ECMO when their general conditions progressively deteriorated to very severe conditions (P/F ratio less than 80 for 24 hours). Compared to previous report, we think the delayed initiation of ECMO may explain their relatively poor prognosis. In addition, *A. Baumannii* infection in these patients after ECMO therapy indicated a cause death due to secondary infection.

Steroid has not been recommended for influenza treatment, and some researches showed even disadvantage of using steroid in these patients [32], [33]. In this study, due to the first time recognizing H7N9 infection, moderate dosage of steroid has been applied with tight restrictions (Table 1). We increased the dosage (40 mg Qd to 40 mg Bid or 80 Bid) when (i) patients displayed worse conditions; (ii) 50% increase of lung infiltration was found; or (iii) CRP increased more than 50% within 24 hours. Mechanical ventilation with low tidal volume and PEEP (10–18 cmH_2_O) was also applied but treatment efficacy could not be estimated at a lack of control groups. Because we do not have adequate control patients comparing patients who had steroid use, it is not clear whether steroid is beneficial or not in H7N9 infected patients in our studies. Based on previous studies in H1N1 patient, it seems that steroid may not provide additional benefit.

In summary, the clinical characteristics of 18 H7N9 patients have been analyzed to unveil possible prognostic indicators. Routine blood test and blood chemistry might be useful in monitoring disease progression and predicting prognosis. Certain laboratory tests have predicted patients' mortality such as plasma CRP while virus positive time was significantly associated with poor outcome, suggesting that virus antibody conversion was critical for recovery from H7N9 infection. On top of that, H7N9 was compared with H5N1 and H1N1 to elucidate their similarities and differences; such results might be indicative of diagnosis, treatment and prognosis of influenza pandemics.
