# Correction: Prognosis of 18 H7N9 Avian Influenza Patients in Shanghai

**DOI:** 10.1371/journal.pone.0096591

**Published:** 2014-04-24

**Authors:** The PLOS ONE Staff

The Editor of this article is listed incorrectly. The correct Editor is Jianming Qiu, University of Kansas Medical Center, United States of America. The publisher apologizes for the error.
